# Heartland Virus in Humans and Ticks, Illinois, USA, 2018–2019

**DOI:** 10.3201/eid2607.200110

**Published:** 2020-07

**Authors:** Holly C. Tuten, Kristen L. Burkhalter, Kylee R. Noel, Erica J. Hernandez, Seth Yates, Keith Wojnowski, John Hartleb, Samantha Debosik, April Holmes, Christopher M. Stone

**Affiliations:** University of Illinois at Urbana-Champaign, Champaign, Illinois, USA (H.C. Tuten, K.R. Noel, E.J. Hernandez, S. Yates, C.M. Stone);; Centers for Disease Control and Prevention, Fort Collins, Colorado, USA (K.L. Burkhalter);; Kankakee County Health Department, Kankakee, Illinois, USA (K. Wojnowski);; US Fish & Wildlife Service, Marion, Illinois, USA (J. Hartleb);; Illinois Department of Public Health, Springfield, Illinois, USA (S. Debosik, A. Holmes)

**Keywords:** Phlebovirus, Heartland virus, HRTV, tick-borne diseases, ticks, Ixodidae, *Amblyomma americanum*, Illinois, United States, viruses, vector-borne infections

## Abstract

In 2018, Heartland disease virus infected 2 persons in Illinois, USA. In 2019, ticks were collected at potential tick bite exposure locations and tested for Heartland and Bourbon viruses. A Heartland virus–positive pool of adult male *Amblyomma americanum* ticks was found at 2 locations, 439 km apart, suggesting widespread distribution in Illinois.

Heartland virus (HRTV), a phlebovirus in the order Bunyavirales, is an emerging zoonotic pathogen. In 2009, after 2 cases were identified in persons in Missouri, additional cases were subsequently reported from Kansas, Oklahoma, Arkansas, Missouri, Tennessee, Kentucky, Indiana, Georgia, and South Carolina. Disease onset was most often during April–September (*1*). HRTV symptoms can initially resemble those of ehrlichiosis (*2*) and include fatigue, fever, leukopenia, and thrombocytopenia (*3*). Human illness caused by HRTV infection often requires hospitalization and has resulted in death (*1*).

After 2 persons infected with HRTV in northwestern Missouri reported having noticed attached ticks before symptom onset (*4*), subsequent entomologic studies detected HRTV in nymphal *Amblyomma americanum* ticks. Laboratory studies confirmed the competence of *A. americanum* ticks for transmitting HRTV transstadially and horizontally (*5*). This body of evidence led to the implication of *A. americanum* ticks as the putative vector of HRTV (*2*,*6*). Serologic surveys of mammals and birds subsequently detected HRTV-specific neutralizing antibodies in a variety of mammals, including raccoons and white-tailed deer, suggesting that various medium- and large-sized mammals may serve as hosts (*3*,*7*).

*A. americanum* ticks are vectors of public health concern because of their aggressive biting behavior, willingness to feed on humans, and abundance. Over the past century, their distribution range has expanded northward (*8*), and population establishment continues to increase because of climate change (*9*). Habitat suitability models have suggested that this species’ fundamental niche should reach the center of Illinois (*10*) or eventually encompass the state entirely (*9*).

In July 2018, a Kankakee County, Illinois, resident (case-patient 1) reported having incurred multiple tick bites while camping on private residential property. The patient was hospitalized with fever, headache, myalgia, nausea, diarrhea, and a diffuse maculopapular rash. In September 2018, a Williamson County, Illinois, resident (case-patient 2) noticed tick bites while staying at a campground near home. The patient was hospitalized with fever, headache, myalgia, fatigue, decreased appetite, nausea, and diarrhea. The Centers for Disease Control and Prevention (CDC) confirmed that clinical samples from both patients were positive for HRTV. We subsequently performed entomologic investigations to determine tick density and HRTV prevalence among tick populations at the likely sites of exposure. 

## The Study

The suspected sites of human exposure were determined according to case-patient interviews conducted by local county health departments (Figure). Two of the 3 sites were in an area considered endemic for *A. americanum* ticks, and the other site was near the putative current northern distribution range limit for this tick vector.

**Figure:**
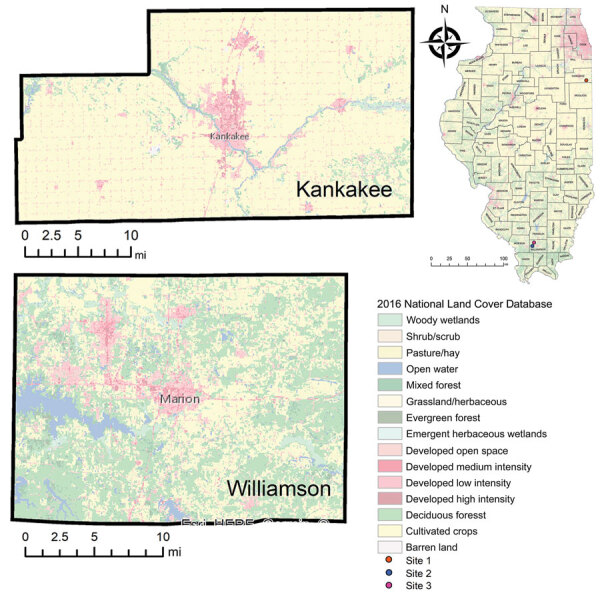
Tick collection sites associated with 2 cases of Heartland virus infection in humans, Kankakee and Williamson Counties, Illinois, USA, 2019. Locations of the counties are indicated by red dots on the Illinois map.

For case-patient 1, the potential exposure site was an ≈40-acre rural homestead in Kankakee County, which had an assemblage of barnyard animals, including chickens, goats, horses, and turkeys (site 1) and a small amount of forest surrounded by extensive cropland. For case-patient 2, in Williamson County, a potential exposure site consisted of 2 adjacent lakeshore campgrounds located within a heavily wooded wildlife refuge (site 2) and another was a suburban home with sparse tree cover (site 3). We observed deer at site 1 during collection visits on June 21 and 25, 2019, and deer, coyotes, and racoons at site 2 during visits on July 11 and 12, 2019. A pet dog lived at the residence at site 3, which we visited on July 11, 2019.

We collected ticks by dragging along 150-m transects (sites 1 and 2) and with carbon dioxide traps consisting of a 1 m^2^ white cloth laid on the ground with 0.5 kg of dry ice left in the center to sublimate for 2 hours before returning to collect ticks (sites 1–3). We collected live ticks into 14-mL plastic centrifuge tubes (TPP, https://www.tpp.ch) that had been modified by applying carpet tape between the lid and tube mouth. We added ticks through a tape-covered hole punched in the center of the paper-backed side of the tape; the sticky side of the tape facing the tube interior immobilized the ticks before they could exit, enabling their secure transport while alive (Video**).** Ticks were either kept alive (site 1) or killed in the field at the end of the day and kept on dry ice (sites 2 and 3) during transport to the Illinois Natural History Survey Medical Entomology Laboratory (Champaign, IL, USA), where they were identified and sorted by species, life stage, and sex (*11*,*12*) on a chill table and maintained at −80°C. Ticks were then shipped on dry ice to the CDC Arboviral Diseases Branch (Fort Collins, CO, USA) for Heartland and Bourbon virus testing, where tick pool homogenization, RNA extraction, and virus screening were performed by real-time PCR as previously described (*2*,*13*). The prevalence of virus infection from pooled samples was calculated by using PooledInfRate, which implements a bias-corrected maximum-likelihood estimation method (*14*).

**Video:** Adult female and male and nymphal *Amblyomma americanum* ticks being transported alive in field within a secure tube.

A total of 70 pools of adult ticks and 23 pools of nymphs were tested (Table 1). The median pool size for adult ticks was 10 (range 1–10) and for nymphs was 30 (range 3–33). A single pool of male *A. americanum* ticks from each county was positive for HRTV (cycle threshold values of 21.7 for site 1 and 24.1 for site 2 by first PCR, 23.2 and 25.3 after confirmation by second PCR); Bourbon virus was not detected. The estimated prevalence of HRTV in adult male *A. americanum* ticks was 9.46/1,000 ticks at site 1 and 7.60/1,000 ticks at site 2 (Table 2).

**Table 1:** Collection methods and number of ticks of each species and life stage collected in 2 counties, Illinois, USA, 2019

Site, method, tick species	Stage	Sex	No. collected	Density/1,000 m^2^
Site 1*			659	
Dragging				
* Amblyomma americanum*	Adult	F	93	26
	Adult	M	90	25
	Nymph	Not applicable	338	93
* Dermacentor variabilis*	Adult	F	15	4
	Adult	M	10	3
Carbon-dioxide trap				
* A. americanum*	Adult	F	18	Not applicable
	Adult	M	17	Not applicable
	Nymph	Not applicable	75	Not applicable
* D. variabilis*	Adult	F	1	Not applicable
	Adult	M	1	Not applicable
* Ixodes scapularis*	Nymph	Not applicable	1	Not applicable
Site 2†			498	
Dragging				
* A. americanum*	Adult	F	32	15
	Adult	M	44	21
	Nymph	Not applicable	159	76
* D. variabilis*	Adult	F	1	0.5
	Adult	M	2	1
Carbon-dioxide trap				
* A. americanum*	Adult	F	118	Not applicable
	Adult	M	88	Not applicable
	Nymph	Not applicable	48	Not applicable
				
* D. variabilis*	Adult	F	3	Not applicable
	Adult	M	3	Not applicable
Site 3‡			9	
Carbon-dioxide trap				
* A. americanum*	Adult	F	4	Not applicable
	Nymph	Not applicable	4	Not applicable
				
* D. variabilis*	Adult	F	1	Not applicable

*Site 1, Kankakee County, visited June 21 and 25, 2019; dragging, n = 24 × 150 m transects; carbon dioxide traps, n = 3. †Site 2, Williamson County, visited July 11–12, 2019; dragging, n = 14 × 150 m transects; carbon dioxide traps, n = 9. ‡Site 3, Williamson County, visited July 11–12, 2019; no dragging performed because of site size; carbon dioxide trap, n = 2.

**Table 2:** Prevalence of Heartland virus in ticks, by location, species, and sex in 2 counties in Illinois, USA, 2019*

Species	Stage	Sex	County	No. ticks collected	No. pools	No. positive pools	Infection rate/1,00 ticks, MLE (95% CI)
*Amblyomma americanum*	Adult	M	Kankakee	107	16	1	9.46 (0.55–46.1)
*A. americanum*	Adult	F	Kankakee	111	12	0	0 (0–29.5)
*A. americanum*	Nymph	NA	Kankakee	413	15	0	0 (0–8.2)
*A. americanum*	Adult	M	Williamson	132	15	1	7.6 (0.44–36.9)
*A. americanum*	Adult	F	Williamson	154	17	0	0 (0–22.16)
*A. americanum*	Nymph	NA	Williamson	211	8	0	0 (0–14.5)
*Dermacentor variabilis*	Adult	Both	Kankakee	27 (16 F, 11 M)	4	0	0 (0–92.8)
*D. variabilis*	Adult	Both	Williamson	10 (5 F, 5 M)	6	0	0 (0–248.8)

*MLE, maximum-likelihood estimation; NA, not applicable.

## Conclusions

One year after 2 cases in humans were detected, HRTV was detected in *A. americanum* ticks collected from the suspected exposure locations in Illinois. Because of abundant suitable habitat and established *A. americanum* tick populations (*10*), it is notable but predictable that this pathogen emerged in southern Illinois. The density of and HRTV detection in *A. americanum* ticks at the northern edge of their distribution range in Kankakee County was unexpected. Our findings suggest that *A. americanum* ticks are established along their northern distribution range at high densities. Consequently, diseases associated with *A. americanum* ticks must be on the radar of physicians and public health officials throughout Illinois.

Detection of HRTV in adult *A. americanum* ticks suggests that infected ticks may have overwintered in the area and maintained HRTV infection transstadially. The presence of HRTV in adult male, but not female or nymph, ticks was also reported in a study in Kansas, where the infection rate varied from 3.29 to 8.62/1,000 ticks (*15*), similar to our findings. Additional tick collection efforts and wildlife serosurveys will help assess whether transmission cycles are active in Illinois and enhance our knowledge of the transmission ecology of this rare pathogen.
