# Supplementary figures and images for: Heartland Virus in Humans and Ticks, Illinois, USA, 2018–2019
Source: Emerg Infect Dis. 2020 Jul;26(7):1548–52. doi: 10.3201/eid2607.200110 (PMC7323525; doi:10.3201/eid2607.200110)

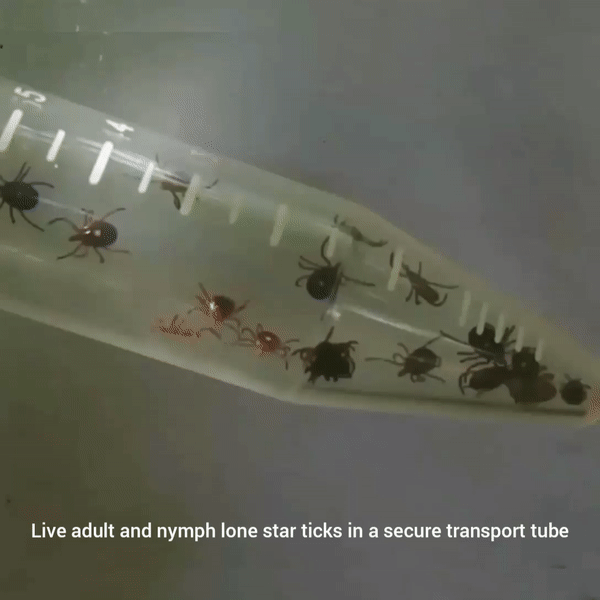

Supplement: Supplementary file 1 [file 20-0110-V.gif]
